# Causal Effect of Character Gender on Readers’ Preferences

**DOI:** 10.63744/hef2punudmph

**Published:** 2025-12

**Authors:** Federica Bologna, Ian Lundberg, Matthew Wilkens

**Affiliations:** 1 Department of Information Science, Cornell University, Ithaca, NY 14850; 2 Department of Sociology, University of California, Los Angeles, CA 90095

**Keywords:** gender, readership, randomized experiment, survey

## Abstract

Stakeholders in the publishing industry observe strong gender homophily between authors and readers, but it is unclear what causes this alignment. One possible mechanism involves reader preferences for gendered characters – specifically, the largely untested hypothesis that men are less inclined to read books with women protagonists, while women are more willing to read stories featuring protagonists of any gender. This mechanism may affect not only the publication but also the promotion and adaptation of books with women characters. However, there is little causal evidence about the underlying claim: for a given book, would men be more likely to read it if it featured a man character? Would women be more likely to read it if it featured a woman character? Our study provides new causal evidence on these questions in a well-powered study. Using a randomized survey experiment involving 3,000 participants, we isolate the causal effect of protagonist gender on reader preferences. Our findings reveal that the effect of character gender is close to zero. Contrary to popular belief, men’s reading preferences were unaffected by the gender of the protagonist. Women, on the other hand, displayed a slight preference for stories featuring women protagonists. These results challenge a subset of industry assumptions and reassure publishers and authors that books about women characters will not necessarily alienate readers.

## 1 Introduction

Literary fiction was long dominated by men. From the early nineteenth century until very recently, most published novels were written by men [28]. Books by men devoted the majority of their narrative attention to male characters, while books by women were more equally balanced [15; 29]. These asymmetries produced a literary marketplace that supplied far more stories of men’s experiences than of women’s. Similar discrepancies have existed in book reviewing (mostly performed by men on books authored by men that told stories about men) and in the distribution of literary prizes [2; 17; 18; 20; 23; 27].

In recent years, the gender composition of published literature has shifted significantly. Women now make up the majority of published authors and women are more likely to read books—and to read them intensively—than are men [30]. These shifts have provoked anxiety among a subset of male authors and other commentators that literary fiction has become a “largely female pursuit,” with purportedly deleterious effects on boys’ academic achievement and on men’s civic engagement [1; 7; 8; 19].

Most analysis of gender and literary publishing has focused on the relatively strong gender assortment between authors and readers. But it is unclear why men are more likely to read books by men and women books by women [9; 24]. One potential causal mechanism is an assumed but understudied asymmetry between men and women as readers, namely that men prefer to read stories *about* men, while women are comparatively indifferent to the gender of the characters in the books they read. If this assumption is correct, it incentivizes a literary mix enriched in stories about men relative to the gender composition of readership, since any individual book could maximize its potential readership by centering male characters.

There is limited evidence that children prefer stories about characters who share their gender identity or other aspects of their social self-perception, even as children’s books (like their adult counterparts) remain enriched in stories about men and boys [12]. While [16] found no statistically significant differences in fourth-grade students’ reading behavior based on protagonists’ gender, both [3] and [4] find that adolescents and fifth-grade students prefer stories with protagonists of their own gender identity. [14] argues that, although boys and girls like men protagonists more overall, protagonist’s age is a more powerful variable in relation to story preference than is gender.

Existing research on character gender and adults’ reading preferences is scarce and limited. In an interview study of 29 men and 29 women, [26] finds that the majority of men prefer books with men protagonists, whereas the majority of women express no preference between men and women protagonists. On the other hand, [5] finds that, among 110 college students, both men and women prefer stories with men protagonists.

It is thus unclear whether and to what extent readers’ preferences are influenced by the ascribed gender of the characters they encounter. To address this commercially and socially important gap in knowledge, we address the existence, strength, and polarity of gender preferences among readers by measuring the causal effect of varying protagonist gender on readers’ desire to continue reading a short story.

## 2 Data and methods

In order to measure the average causal effect of character gender on reader preferences we employ a paired-choice vignette experiment with forced choice. Participants read two stories, one with a woman character and one with a man character, and are asked to choose which one they would continue reading. We randomize which story features the woman character to control for confounding effects due to participants’ preferences for specific narrative themes.

Our design is similar to a broad literature on vignette factorial surveys and conjoint experiments [11; 31], in which people choose between a pair of options with attributes (e.g., gender) that have been randomized. These designs capture human preferences that align with those observed in real-world decisions [10]. Our design is a particularly simple case: gender is randomly assigned across a pair of stories presented in random order. This design identifies the causal effect of protagonist gender among a pair of stories.

We recruit 3000 people currently residing in the United States from the crowd-sourcing platform Prolific. Using Prolific’s pre-screening feature, we select 1500 women and 1500 men. However, this pre-screening can be imprecise, as gender identity is self-reported and may change from the pre-screen values recorded in Qualtrics to the report the respondent provides in our survey. We operationalize gender as the respondent’s self-report within our survey. We exclude participants of other gender identities from our experiment due to the inability to collect a satisfactory quantity of reliable data about them using Prolific. Consequently, we remove 17 responses from participants who self-report other gender identities (8 in the women’s response collection and 9 in the men’s response collection). We obtain a total of 2983 responses (W = 1492, M = 1491).

### 2.1 Experimental design

After consenting to participate in the study, respondents are shown two stories, each about 500 words in length. Due to their length, the stories are displayed in succession over two different web pages. One story is about a hike, the other is about a coffee shop. One story, selected at random, features a woman character (as indicated by the pronouns “she” and “her”) and the other features a man character (“he”/”him”). We do not modify the names of the characters but instead assign names without strong gender associations to both stories: Sam for the protagonist of the hike story, and Alex for the protagonist of the coffee shop story. To control for confounding due to topic preferences and display order, we randomize both which story features the woman character and which story appears first. Respondents are then required to answer correctly four reading comprehension questions about the stories to continue the study. This ensures that the participant processed and understood the stories. After the comprehension check, respondents are asked to express their reading preference by choosing which story they would continue reading. Finally, participants respond to a few questions to collect demographic data. The full text of the stories and of the survey is available in Appendix A.

### 2.2 Outcomes

After reading the two assigned stories and completing the reading comprehension check, we ask respondents the following question: “Suppose you had both novels in your hand. Which one would you continue reading?” Participants have the option to answer “Novel A” or “Novel B,” and are required to supply an open-ended explanation of their choice (minimum length 200 characters).

### 2.3 Demographics

We collect the respondents’ self-reported age, gender, family income, country of origin, US zip code, and political orientation. For the complete text of these questions, see Appendix A. The demographic characteristics of our sample are typical of online samples: 66% are age 45 or younger, 51% describe themselves as liberal or very liberal compared with 22% conservative or very conservative, and 68% report incomes below $100,000 per year. While evidence from our online sample may not generalize to other populations, a countervailing strength of this sample is the high statistical power that comes from our ability to recruit many respondents online.

### 2.4 Causal identification

Let *Y*_*i*_ indicate whether the respondent chose the hiking story. Let *A*_*i*_ = Woman versus *A*_*i*_ = Man indicate the gender of the protagonist in the hiking story that was randomly assigned to respondent *i*. Let $Y_{i}^{\text{Woman}}$ and $Y_{i}^{\text{MAN}}$ be potential outcomes indicating whether respondent *i* would choose the hike story if it was about a woman or a man, respectively. We observe $Y_{i}^{\text{Woman}}$ when *A*_*i*_ = Woman and $Y_{i}^{\text{MAN}}$ when *A*_*i*_ = Man. Because the treatment *A*_*i*_ is randomized, the expected potential outcome if assigned to a condition equals the expected observed outcome among those factually assigned to that condition, *P*(*Y* ^*a*^ = 1) = *P*(*Y* = 1 | *A* = *a*). We report the probability of choosing the hiking story if it is about a woman and if it is about a man, and the causal effect that is the difference of these two estimates. We estimate separately by respondent gender.

## 3 Results

Against the expectations of many publishing industry stakeholders, we do not find evidence that men readers have a preference for men characters. In our experiment, the effect of character gender among men participants is close to zero. Men participants show a 76% probability of choosing the hike story when it features the woman character and a 75% probability of choosing the hike story when it does not feature the woman character. Being randomly assigned to have a woman protagonist in the story thus increases men’s probability of choosing the story by 0.008 [CI −0.036, 0.051]. This result is not statistically distinguishable from zero.

Women participants show a slightly stronger preference for a story with a woman protagonist. We find a 77% probability of choosing the hike story when it features a woman character compared with a 70% probability when it does not feature the woman character. The estimated treatment effect for women is small but significantly positive, at 0.062 [CI 0.017, 0.106].

While participants show an overall preference for the hike story over the coffee story, this fact does not influence our results. Since participants were randomly assigned to reading the hike story with either the woman or man character, the preference for the hike story does not confound our estimation of the effect of character gender on reader preferences.

## 4 Discussion

The strong correlation between author and reader gender has often been attributed to the assumption that male readers prefer stories with male protagonists. To test the validity of this belief, our study randomly assigns protagonist gender across a pair of stories and estimates its effect on reader preferences. By isolating the causal effect of protagonist gender, we produce a surprising result: protagonist gender has almost no effect on reader preferences.

Contrary to previous qualitative or lower-powered studies [5; 26], we do not find evidence of a strong preference for either same-gender characters or for men characters by adult readers. Instead, we find a small but non-zero effect of character gender on reader preferences, such that readers (particularly women) show a slight preference for stories randomized to have a woman protagonist.

While our results are limited to this particular pair of stories, they are powerful because the stories are fixed and only the gender of the protagonist is randomized. By showing that having a woman protagonist does not reduce men’s inclination to read a story in this setting, we undermine the pervasive belief that men might be alienated from reading as the representation of women and girls as protagonists increases [1; 7; 8; 19]. Our results suggest that participants in the publishing ecosystem need not assume that writing about women will cost them their audience, and fiction editors should let go of their reservations about publishing books with woman protagonists.

## 5 Limitations and future work

Our results leave some unanswered questions, such as why men might read more stories by men, which predominantly feature men characters [9; 15; 29]. This might be the case, for example, if men and boys tend to read genres (e.g., mysteries) in which men and boys are over-represented as protagonists. Our results suggest that if an author in one of these genres replaced a man protagonist with a woman protagonist, then men might still read that book at the same rate. Genre is held constant in our study; future work that randomizes genre would be needed to understand how the genre of writing affects men’s and women’s reading habits.

Similarly, a second open question is why men and boys read less in general than women and girls. Our results are compatible with the hypothesis that men’s comparatively low rates of reading are caused not by character gender but by gendered expectations and socialization processes that discourage boys from reading at rates equal to girls [13; 21; 22; 33].

Future work could also investigate whether other features of literary texts, such as author gender or gender-conformity, may affect reader behaviors. Scientific work on these features is paramount: men reviewers and readers read more books by men than by women [2; 9; 25]. There is preliminary evidence that boys are more interested in reading about girl characters when those characters engage in activities usually ascribed to boys [6; 32]. No evidence is available regarding the effect of gender conformity in the case of adult readers.

In addition, future work could include readers of other gender identities. One of the limitations of this study is the exclusion of gender-nonconforming participants and characters from the study population due to the scarcity of available data on this group. These readers may exhibit different reading behaviors in relation to character gender than what we have observed in this study.

Lastly, this study utilized unpublished short stories created specifically for this experiment. Future research could explore whether reading preferences change in the case of excerpts from published fiction. However, using well-known texts in an experimental setting may be infeasible. If a participant were randomly assigned to read, for example, a modified passage from *Harry Potter* featuring Sally Potter, the participant would be able to guess the purpose of the study, potentially biasing their responses. Our use of unpublished short stories limits our external validity but aids strong internal validity by reducing participants’ suspicion about the study’s objectives.

While this study could not investigate all the possible interactions between gender and fiction readership, we hope that our findings will inspire researchers, readers, and authors to engage with the expanding universe of women characters. One need only think about the past to recognize that both men and women can connect with powerful women characters, from Celie in *The Color Purple* to Katniss Everdeen in *The Hunger Games*, from Circe in the eponymous book to June in *The Handmaid’s Tale*. Demonstrating that men want to read about women characters is only a first step toward a more robust and equitable publishing industry.

## Figures and Tables

**Figure 1:**
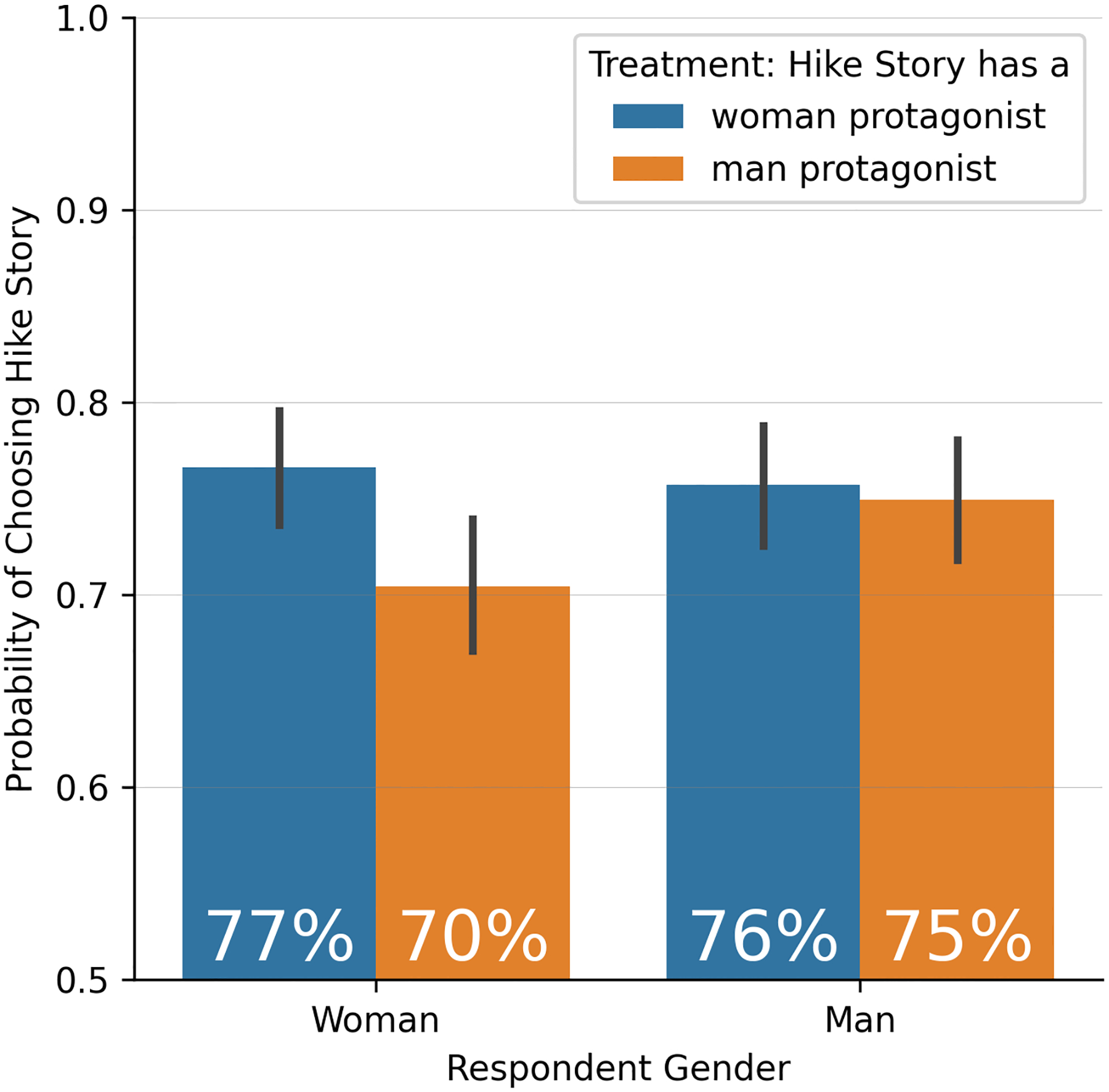
Probability of choosing the hike story within treatment conditions and subgroups.

**Figure 2:**
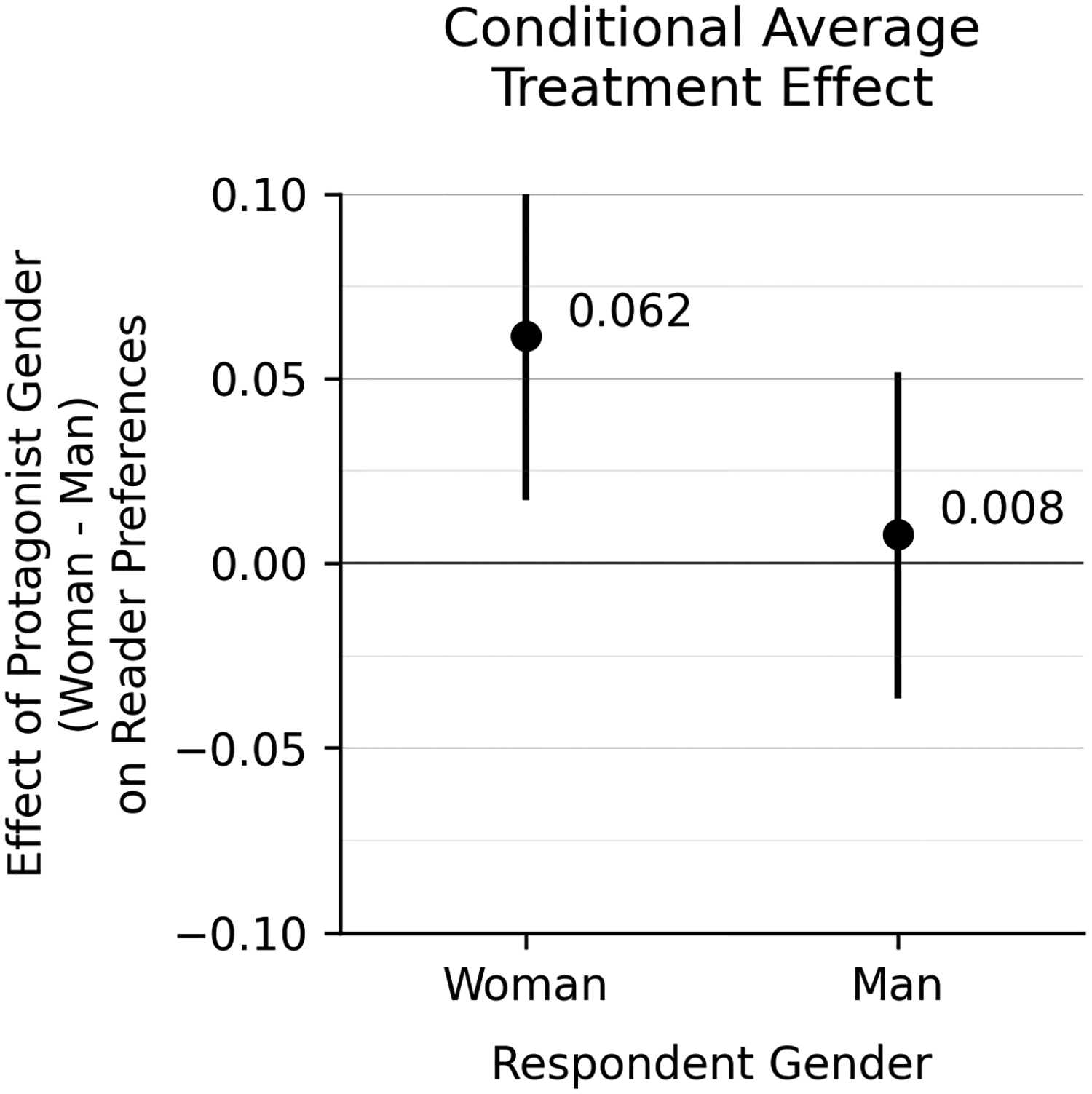
Conditional average treatment effect. Each estimate is a difference across the bars in Figure 1.

## A Appendix

### A.1 Full text of the stories

#### A.1.1 1.1 Hike story

Sometimes Sam wondered who was in charge of **her/his** life. The Sam of the present, or the imagined one that wakes up naturally at dawn and eats all of the greens in **her/his** fridge? Five alarms came and went before Sam rose from **her/his** plush covers. *Damn, it’s already going to be hot by the time I get to the trailhead*. **She/He** quickly ate breakfast, got ready, and left.

The door to **her/his** Subaru was hot to the touch. *Who moves to Tucson with a black car?* Sam had taken the day off of work, so **she/he** took **her/his** time enjoying the scenic vistas of the desert. All of which were so unlike the lush, temperate rain forests of Washington. As **she/he** arrived at the trailhead, **she/he** inspected the map. *Christ, six miles?* Sam looked to the trail, which had a slight incline at the base, but was for the most part flat. *Fine! I guess I took the day off for a reason…*

Sam took frequent water breaks, which wasn’t too embarrassing, since **she/he** saw only one other hiker on the trail. It also gave **her/him** time to take in the diversity of cacti. Shade was in short supply but, according to the map, where a stream connected with the trail was a stand of juniper trees where **she/he** could take a longer rest.

The sounds of the stream greeted **her/him** well before the sight of it did. **She/He** tossed **her/his** bag to the ground and slumped down against the bark of a juniper. Once **she/he** downed some water and gave **her/his** eyes a rest, **she/he** turned **her/his** gaze to the running water. Sam moved from the shade to the bank and ran **her/his** hand through the water, inspecting the reflections.

Sam returned to the tree and ruffled through the rucksack. At the very bottom, crushed and with light tearing, was a letter. Above **her/his** old address in Olympia was a note, not a name. *Read me at the end*. The backside of the letter had been opened, but the note inside remained crisp. *I guess it’s time, isn’t it?*

#### A.1.2 Coffee story

A twinge from **her/his** calf is what jostled **her/him** awake. *I really should be more careful jumping around the snow all willy-nilly*. Alex began massaging **her/his** calf and winced as he hit a bruise. The clock read just after six-thirty, so the cafe would be open. He splashed **her/his** face with water, quickly put on a hand-knit sweater and a pair of snow boots, and made **her/his** way out of the door.

The snow was in between soft, silent powder and settled, crunchy ice. Each step was a nice surprise, not unlike the fallen leaves of autumn. A burly man was out front of the cafe, shoveling the snow along the uneven sidewalk. Alex gave a nod to the man before pulling open the worn, wooden door.

Alex removed a small sketchbook and nondescript pen from **her/his** coat pocket and opened to an empty page. *Wow, the snow is really coming down now,* he mused, turning back to the cafe. *I guess I am here for at least a few hours*. Directly opposite **her/him** was an empty wooden chair with surprisingly complex upholstery patterning. He began sketching the scene before **her/him**.

First, simple outlines of the objects to get the scale right. While he should have taken more time to get those details down, he was obsessed with the chair and its craft. How the armrests had deep, symmetrical, swirling designs and bulbous flares where you rest your hands. *It needs…something*. He pulled out a small deerskin pouch from **her/his** coat. *This!* Where once only smooth black lines had lived, color now erupted. Magenta, royal gold, and vibrant turquoise.

Pausing only to sip **her/his** espresso, the drawing tunnel vision had allowed time to pass and the sun to make itself apparent. As Alex lifted the notebook up in a ray of sunshine, a slip of paper fell to the floor. Alex picked it up, *Shoot! This is happening today, isn’t it?* On the paper was a call for submissions to an art gallery. One he was too intimidated to submit to. Wired from espresso, Alex slammed **her/his** notebook closed, packed **her/his** supplies, and threw **her/his** coat back on.

### A.2 Survey

#### A.2.1 Consent

##### Welcome to our study!

Thank you so much for participating in this brief survey to improve the understanding of reader preferences.

This survey requests personal information limited to age, gender, location, income and political orientation. We estimate that this survey should take at most 10 minutes to complete.

Please read this page carefully and check the box at the bottom of the page if you agree to participate.

##### You must be 18 years old or older to participate.

###### Who we are

This study is conducted by researchers from Cornell University: Federica Bologna, Ian Lundberg and Matthew Wilkens.

###### What the study is about

This survey is aimed to improve our understanding of readers’ preferences and which elements of the first few pages of a novel motivate the reader to continue reading.

###### What we will ask you to do

If you agree to participate, you will be asked to complete a brief survey regarding your reading preferences. You will be expected to read the beginning of two novels, answer a few questions on the passages provided, and provide information on your age, gender, location, income, and political orientation.

##### Compensation

You will be compensated with a $2.50 monetary reward for participation in the survey.

##### Risks

We anticipate that your participation in this survey presents no greater risk than everyday use of the Internet. However, if any of the survey questions cause you fatigue, discomfort, or upset, you are welcome to discontinue at any time and we encourage you to do so.

##### Rights

Taking part in this study is completely voluntary. If you decide to participate, you are free to withdraw at any time for any reason. However, should you decide to withdraw, you will not be eligible for the $2.50 monetary reward.

##### Benefits

There may be no direct or indirect benefit to you from participating in this study. However, the insights obtained from this research could lead to the improvement of reader experiences.

##### Privacy

Your responses will be completely anonymous. We will not collect your name or any other personally identifiable information. Your responses will be anonymized and stored in an encrypted database. Only researchers involved in this study will have access to this information. De-identified data from this study may be shared with the research community at large to advance scientific knowledge. We will remove any personal information that could identify you before files are shared with other researchers to ensure that, by current scientific standards and known methods, no one will be able to identify you from the information we share.

###### If you have questions about the study

You may contact the Cornell research team by emailing Federica Bologna at fb265@cornell.edu. If you have concerns regarding your rights as a participant, you may contact the Institutional Review Board (IRB) for Human Participants at 607-255-5138 or access their website at http://www.irb.cornell.edu. You may also report your concerns or complaints anonymously through Ethicspoint online at www.hotline.cornell.edu or by calling toll free at 1-866-293-3077. Ethicspoint is an independent organization that serves as a liaison between the University and the person bringing the complaint so that anonymity can be ensured.

If you agree to these conditions, please click “I consent to participate” below. If you do not agree, click the “I do not consent to participate” option.

By agreeing to participate, you confirm that you are over 18 years of age.

I have read the above information.

- I consent to participate.
- I do not consent to participate.

#### A.2.2 Prolific ID

What is your Prolific ID?

Please note that this response should auto-fill with the correct ID.

[*Short answer text box here*]

#### A.2.3 Introduction

In order to better understand readers’ preferences, we would like you to carefully and attentively read two passages.

After the two passages, you will be asked to respond to four reading comprehension questions. These questions are meant to confirm that you read the passages.

If you answer them incorrectly, you will have the opportunity to read the stories and answer the comprehension questions two more times. Once you will answer the questions correctly, you will be able to complete the survey.

#### A.2.4 First story

These are the first pages of Novel A. Please read carefully.

[*Full story here*]

#### A.2.5 Second story

These are the first pages of Novel B. Please read carefully.

[*Full story here*]

#### A.2.6 Reading comprehension

##### If first story is the hike story:

The following 4 reading comprehension questions are meant to confirm that you carefully read the previous passages before you can complete the survey. If you answer incorrectly, you will still have two more opportunities to read the passages, answer the comprehension questions and complete the survey.

In Novel A, how long is the trail that Sam takes?

- 3 miles
- 8 miles
- 6 miles

In Novel A, what plant does Sam rest against?

- Juniper tree
- Prickly pear
- Saguaro

In Novel B, when does Alex go to the cafe?

- Afternoon
- Evening
- Morning

In Novel B, what is the material of the chair Alex sketches?

- Metal
- Wood
- Plastic

##### If first story is the coffee story:

The following 4 reading comprehension questions are meant to confirm that you carefully read the previous passages before you can complete the survey. If you answer incorrectly, you will still have two more opportunities to read the passages, answer the comprehension questions and complete the survey.

In Novel A, when does Alex go to the cafe?

- Afternoon
- Evening
- Morning

In Novel A, what is the material of the chair Alex sketches?

- Metal
- Wood
- Plastic

In Novel B, how long is the trail that Sam takes?

- 3 miles
- 8 miles
- 6 miles

In Novel B, what plant does Sam rest against?

- Juniper tree
- Prickly pear
- Saguaro

#### A.2.7 Incorrectly answered questions

[*If any questions were answered incorrectly, show the following section:*]

You answered the following questions incorrectly:

[*List of questions that were incorrectly answered*]

You will be shown the two passages again. You will have the opportunity to re-read them and to answer the comprehension questions another time.

#### A.2.8 Rating

Suppose you had both novels in your hand. Which one would you continue reading?

- Novel A
- Novel B

Your opinion is fundamental to understand reader preferences. Please take your time to answer this question. In your own words, briefly share your motivation for your choice (minimum 200 characters, or about 40 words).

[*Long answer text box here*]

#### A.2.9 Demographics

This section has the purpose of getting to know you a little better. This will allow us to connect readers’ preferences with their lived experiences and contextualize our results. It is important to us that you answer these questions honestly.

Your age:

- 18–24
- 25–35
- 36–45
- 46–55
- 56–65
- 65+
- Prefer not to disclose

Your gender:

- Woman
- Man
- Non-binary
- Prefer to self-describe: [*Short answer text box here*]
- Prefer not to disclose

What was your total family income before taxes during the past 12 months?

- Less than $25,000
- $25,000–$49,999
- $50,000–$99,999
- $100,000–$199,999
- More than $200,000
- Prefer not to disclose

What is your country of origin?

[*Standard list of countries from Prolific*]

Do you currently reside in the United States?

- Yes
- No
- Prefer not to disclose

What is your current United States Zip Code? [*Only if answered yes to above question*]

[*Short answer text box here*]

How would you describe your political views?

- Very conservative
- Conservative
- Moderate
- Liberal
- Very liberal

#### A.2.10 End of survey message

We thank you for your time spent taking part in this study. Please click the button below to be redirected back to Prolific and register your submission.
